# Optimal control for stochastic neural oscillators

**DOI:** 10.1007/s00422-025-01007-3

**Published:** 2025-03-20

**Authors:** Faranak Rajabi, Frederic Gibou, Jeff Moehlis

**Affiliations:** Department of Mechanical Engineering, University of California, Santa Barbara, Santa Barbara, CA USA

**Keywords:** Stochastic optimal control, Neuronal desynchronization, Hamilton–Jacobi–Bellman equation, Computational modeling

## Abstract

This study develops an event-based, energy-efficient control strategy for desynchronizing coupled neuronal networks using optimal control theory. Inspired by phase resetting techniques in Parkinson’s disease treatment, we incorporate stochasticity of the system’s dynamics into deterministic models to address neural system intrinsic noise. We use an advanced computational solver for nonlinear stochastic partial differential equations to solve the stochastic Hamilton–Jacobi–Bellman equation via level set methods for a single neuron model; this allows us to find control inputs which drive the dynamics close to the system’s phaseless set. When applied to coupled neuronal networks, these inputs achieve effective randomization of neuronal spike timing, leading to significant network desynchronization. Compared to its deterministic counterpart, our stochastic method can achieve considerable energy savings. The event-based control minimizes unnecessary charge transfer, potentially extending implanted stimulator battery life while maintaining robustness against variations in neuronal coupling strengths and network heterogeneities. These findings highlight the potential for developing energy-efficient neurostimulation techniques with implications for deep brain stimulation protocols. The presented computational framework could also be applied to other domains for which stochastic optimal control problems are prevalent.

## Introduction

Pathological synchronization of neuronal activity is a hallmark of several neurological disorders, including Parkinson’s disease, essential tremor, and epilepsy. This abnormal synchrony is linked to debilitating symptoms such as tremors, rigidity, and impaired motor control, significantly reducing the quality of life for affected patients (Buzsáki 2006; McIntyre and Anderson 2016; Popovych and Tass 2014; Litvak and Pogosyan 2011). In Parkinson’s disease, motor symptoms are strongly correlated with excessive synchronization in the beta frequency band (13–35 Hz) within the basal ganglia-thalamo-cortical network (Little et al. 2013; Arlotti et al. 2018; Oswal et al. 2013; Hurtado et al. 2005). Similarly, in epilepsy, synchronized neuronal firing drives seizure activity, with increased synchronization as seizures spread across neural networks (Engel et al. 2013; Jiruska et al. 2012). Modulating this pathological synchronization is crucial for effective therapeutic interventions like deep brain stimulation (DBS) (Goldberg et al. 2002).

DBS has proven highly effective, especially for movement disorders such as Parkinson’s disease, by delivering continuous high-frequency stimulation (typically >100 Hz) to specific brain areas like the subthalamic nucleus (STN) or globus pallidus interna (GPi) (Lim et al. 2009; McIntyre and Anderson 2016). Despite its success, conventional DBS faces challenges such as diminishing efficacy over time, stimulation-induced side effects, and high energy consumption, necessitating frequent battery replacements (Lempka et al. 2010). Furthermore, its static parameters do not adapt to the dynamic nature of disease symptoms (Arlotti et al. 2018).

Advances in control theory have aimed to address these limitations (Wilson and Moehlis 2022). Closed-loop feedback systems dynamically adjust DBS stimulation based on real-time physiological signals, offering more precise symptom control. For instance, Santaniello et al. developed a minimum variance control system that adjusts stimulation based on the spectral content of local field potentials (LFPs), resulting in better control of tremors (Santaniello et al. 2011). Adaptive DBS (aDBS), which uses biomarkers like beta oscillations in LFPs, personalizes treatment by modulating stimulation in response to symptom fluctuations, enhancing symptom control and reducing energy consumption compared to open-loop DBS (Priori et al. 2013; Yamamoto et al. 2012).

Model predictive control (MPC) and reinforcement learning (RL) are also promising approaches for optimizing closed-loop DBS (CLDBS) for neurological disorders. For example, Haddock et al. (2017) uses MPC with patient-specific models to predict symptoms and optimize stimulation, improving energy efficiency while maintaining therapeutic efficacy. Similarly, RL-based systems can adapt stimulation in real time, achieving energy savings without compromising treatment effectiveness (Gao et al. 2020). These machine learning (ML) techniques are further enhancing aDBS by optimizing stimulation parameters, improving outcomes, and minimizing side effects (Matchen and Moehlis 2021; Watts et al. 2020; Sandoval-Pistorius et al. 2023).

In this study, we build on the work of Nabi et al. (2013), which designed an event-based control strategy to minimize energy use while desynchronizing neuron populations. By incorporating stochastic elements into the control framework, we aim to account for the inherent randomness in neural dynamics. Specifically, we solve the stochastic Hamilton–Jacobi–Bellman (HJB) equation, which governs the optimal control of stochastic systems, to derive energy-efficient control strategies that remain robust in the presence of neural variability.

The stochastic HJB equation provides a robust framework for managing random neural fluctuations in DBS. Backward stochastic differential equations (BSDEs) were introduced to handle randomness in dynamic systems, forming the basis for this approach (Peng and Pardoux 1992). Further refinements were made with the development of viscosity solutions for the stochastic HJB equation, enhancing the robustness of optimization techniques (Li and Peng 2009). This framework was later extended with the proof of existence and uniqueness of viscosity solutions, making it applicable to practical control problems like DBS (Qiu 2018).

Our computational framework combines stochastic optimal control theory with numerical solutions of the HJB equation, a nonlinear partial differential equation that characterizes the optimal control strategy. This mathematical framework enables us to compute optimal control policies for single neurons, which we then apply to neural networks. The event-based implementation of these control policies, where stimulation is activated only when needed, results in substantial energy savings compared to continuous stimulation approaches.

Our study presents an event-based, energy-efficient control strategy for desynchronizing coupled neuronal networks, leveraging optimal control theory. Building on phase resetting techniques proposed for Parkinson’s disease (PD) treatment, we incorporate system stochasticity into traditionally deterministic models to account for neural system intrinsic noise. To achieve this, we developed an advanced computational solver for solving the stochastic HJB equation.

Our results demonstrate significant desynchronization of neuronal populations through effective randomization of spike timing. Compared to deterministic methods, the stochastic control can achieve notable energy savings. The event-based nature of the control reduces unnecessary charge transfer, potentially extending the battery life of implanted stimulators, while maintaining robustness in the face of variations in neuronal coupling strengths and network heterogeneities.

These findings underscore the potential for developing energy-efficient neurostimulation techniques, with promising implications for deep brain stimulation protocols. The computational framework used also holds potential for broader applications where stochastic optimal control problems are relevant, offering new insights into energy-efficient control strategies in complex systems.

The paper is structured as follows: We begin by introducing the stochastic neuron model in Sect. 2. Section 3 then formulates and solves the optimal control problem using the stochastic HJB equation for a single neuron. Section 4 uses these control inputs with an event-based strategy for desynchronizing populations of neurons, including consideration of the robustness of the results to heterogeneity within the neural population’s coupling. The paper concludes with a summary of our key findings and their implications in Sect. 5.

## Model

Our control inputs will be calculated by considering the following model for a single neuron:

1 $$\begin{aligned} \begin{aligned} \dot{V}&= f_V(V, n) + u(t) + \eta (t), \\ \dot{n}&= f_n(V, n). \end{aligned} \end{aligned}$$

The variables $V$ and $n$ denote the membrane voltage and gating variable for the neuron, respectively. The intrinsic noise $\eta (t) = \sqrt{2D} \mathcal {N}(0,1)$ is modeled as zero-mean Gaussian white noise with variance $2D$. The control input is $u(t) = I(t)/c$, where $I(t)$, measured in $\mu \text {A/cm}^2$, simulates the deep brain stimulation (DBS) input current, and $c = 1 \; \mu \text {F/cm}^2$ is the constant membrane capacitance. The functions $f_V$ and $f_n$ are given by:

2 $$\begin{aligned} f_V= &  \frac{I_b \!- \!\bar{g}_{Na}[m_{\infty }(V)]^3 (0.8 \!-\! n)(V \!- \!V_{Na}) \!- \!\bar{g}_K n^4 (V - V_K) \!- \!\bar{g}_L (V - V_L)}{c}, \end{aligned}$$

3 $$\begin{aligned} f_n= &  a_n(V)(1 - n) - b_n(V)n. \end{aligned}$$

representing the state dynamics of the neuron in the absence of noise and control. This model is a two-dimensional reduction of the well-known four-dimensional Hodgkin-Huxley (HH) model (Hodgkin and Huxley 1952), capturing the essential dynamical behavior of neurons (cf. Keener and Sneyd 1998; Moehlis 2006). The HH model was initially developed for the giant axon of the Loligo squid through experimental studies. The other functions and parameters in this reduced model are

4 $$\begin{aligned} m_{\infty }(V)= &  \frac{a_m(V)}{a_m(V)+b_m(V)} , \end{aligned}$$

5 $$\begin{aligned} a_m(V)= &  0.1(V+40)/(1-\exp (-(V+40)/10)) \;, \end{aligned}$$

6 $$\begin{aligned} b_m(V)= &  4\exp (-(V+65)/18) \;, \end{aligned}$$

7 $$\begin{aligned} a_n(V)= &  0.01(V+55)/(1-\exp (-(V+55)/10)) \;, \end{aligned}$$

8 $$\begin{aligned} b_n(V)= &  0.125\exp (-(V+65)/80) \;, \end{aligned}$$

9 $$\begin{aligned} &  V_{Na}=50 \; \mathrm{{mV}},\; V_K=-77 \; \mathrm{{mV}},\; V_L=-54.4 \; \mathrm{{mV}}, \end{aligned}$$

10 $$\begin{aligned} &  \bar{g}_{Na}=120 \; \mathrm{{mS/cm^2}}, \; \bar{g}_K=36 \; \mathrm{{mS/cm^2}}, \end{aligned}$$

11 $$\begin{aligned} &  \bar{g}_L=0.3 \; \mathrm{{mS/cm^2}}, \; c=1 \; \mathrm{{\mu F/cm^2}}. \end{aligned}$$

Also, $I_b$, measured in $\mu \text {A/cm}^2$, represents the baseline current of the neuron, reflecting the influence of other brain regions. It can be viewed as a bifurcation parameter controlling whether the neuron is in an excitable regime with a stable fixed point and no periodic orbit (for $I_b < 6.36$), a bistable regime with a stable fixed point and a stable periodic orbit (for $6.36\le I_b \le 8.82$), or oscillatory regime with a stable periodic orbit and an unstable fixed point (for $I_b > 8.82$). More detail on the bifurcations for this model can be found in Moehlis (2006). We set $I_b = 10 \; \mu \text {A/cm}^2$ to ensure oscillatory (periodic spiking) behavior, with a spiking period $T_s = 11.85 \; \text {ms}$. The parameters $\bar{g}_{Na}$, $\bar{g}_K$, and $\bar{g}_L$ are the conductances of sodium, potassium, and leakage channels, respectively, while $V_{Na}$, $V_K$, and $V_L$ denote their reversal potentials.

## Optimal control for a single neuron

For (1) with the parameter values given above and in the absence of noise and control, the system has a stable periodic orbit, which we will call the deterministic periodic orbit. It also has an unstable fixed point found numerically to be at $(V_{pl},n_{pl}) \equiv (-59.6,0.403)$, which we refer to as the *phaseless set* because it is the only point in state space for the deterministic system where one cannot define the phase in the sense of isochrons (Winfree 2001). Reference Nabi et al. (2013) found the energy-optimal control stimulus $u_d^*(t)$ that drives a neuron to its phaseless set $(V_{pl},n_{pl})$. The motivation was that then the intrinsic background noise could cause the system to fall on a random isochron, thereby randomizing the phase of the neuron and its next spiking time (Nabi et al. 2013). Notably, the optimal stimulus found in Nabi et al. (2013) was under the assumption that there was no noise, that is, $D=0$.

**Fig. 1:**
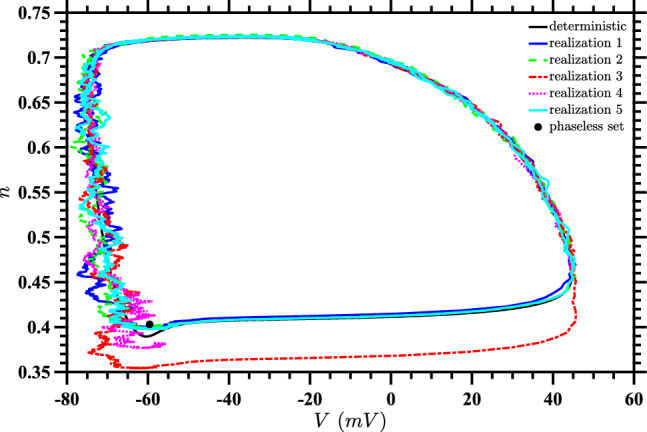
State space diagram for $D=15$ without control input. The deterministic trajectory (black) is compared with five sample stochastic trajectories (colored). All trajectories begin at the same spike point, corresponding to the maximum voltage on the deterministic periodic orbit. The circle indicates the phaseless set for the deterministic system. This highlights how noise disrupts the regular deterministic orbit and causes significant deviations in the system’s behavior

**Fig. 2:**
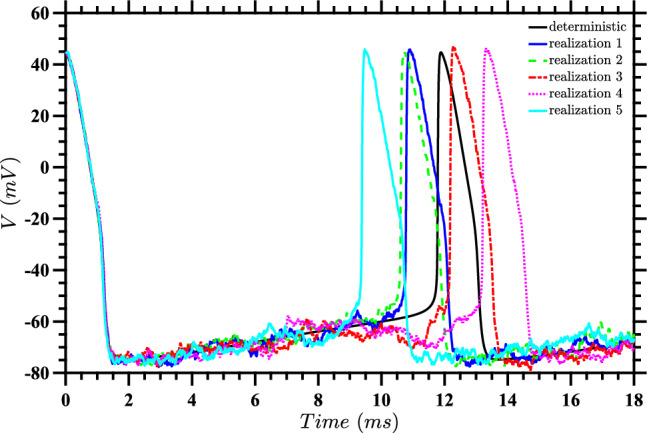
Voltage time series for $D=15$ without control input. These plots show voltage as a function of time for the trajectories presented in Fig. 1. The significant variations in spike timing demonstrate the substantial effect of noise on neural dynamics

In the present paper, we instead find the optimal stimulus to drive the system to the point $(V_{pl},n_{pl})$ when $D \ne 0$, in terms of the expected value for a cost function which captures both the total energy used and proximity to that point. We consider relatively large noise, and will see that this leads to a significant change in the optimal control input with respect to the $D=0$ case considered in Nabi et al. (2013). To get a sense of the effect of noise, we present two figures demonstrating the behavior of a single neuron in the absence of input (i.e., for Eq. (1) with $u(t) = 0$) at a noise intensity of $D=15$. Figure 1 displays a state space diagram comparing the deterministic trajectory with five sample stochastic trajectories. The noise causes significant deviations from the deterministic periodic orbit, leading to substantial fluctuations in neural spike timing, as shown in Fig. 2. These plots demonstrate that noise of this magnitude considerably affects neural dynamics. For all stochastic differential equation simulations presented in this paper, including those shown in these figures, we employed Honeycutt’s second-order stochastic Runge–Kutta method (Honeycutt 1992).

In the following, in order to better stabilize the numerical simulation we will scale down the *V* dimension in (1) by a factor of $K=100$ so that the two states are of same order of magnitude. Consider the change of variables $\textrm{z} \equiv (x,y)=(\frac{1}{K}V,n)$. In view of (1), for a deterministic neuron under control, we get

12 $$\begin{aligned} \begin{aligned} \dot{z}&= F(z) + B u, \end{aligned} \end{aligned}$$

where $$B = [\frac{1}{K}, 0]^\textrm{T}$$ and

13 $$\begin{aligned} \begin{aligned} F(z) = \begin{bmatrix} f_x(z) \\ f_y(z) \end{bmatrix} = \begin{bmatrix} \frac{1}{K}f_V(Kx,y) \\ f_n(Kx,y) \end{bmatrix}. \end{aligned} \end{aligned}$$

This scaling is only for the sake of numerical stability and the results that we present later are all in the original $V-n$ coordinates.

Similar to Nabi et al. (2013), our objective is to find the energy-optimal control law that would take the system (12) to the point $(V_{pl},n_{pl})$ in some pre-specified length of time $[0,T_{end}]$, while minimizing the cost function

14 $$\begin{aligned} J(z,u(t))= &  \int _0^{T_{end}} {u^2} dt + \gamma q(z(T_{end})). \end{aligned}$$

The first term in this cost function characterizes the total input energy being used, and the second term is an end-point cost, ${\gamma }q(z(T_{end}))$, that captures proximity to the phaseless set. The parameter $\gamma$ is a penalizing scalar. We consider bounded inputs, i.e., $|u|\le u_{max}$, as would be the case in practice due to hardware limitations as well as tissue sensitivity. Reference Nabi et al. (2013) showed that this could be done by solving the appropriate HJB equation for what we will call the deterministic cost-to-go function $\mathcal {V}_d(z,\tau )$, which gives the minimum cost that one needs to pay to go from $(z,\tau )$ to the end point subject to the constraints on the optimal control and with no noise, i.e., $D=0$ (Kirk 1970; Hespanha 2007).

For the present problem with $D \ne 0$, with the same scaling we get

15 $$\begin{aligned} \dot{z} = F(z) + B u(t) + B \eta (t). \end{aligned}$$

This can be rewritten in the Ito sense as

16 $$\begin{aligned} d z(t) = (F(z(t)) + B u(t)) dt + B \sqrt{2 D} dW, \end{aligned}$$

where *W*(*t*) is a standard Wiener process. We will solve for the *stochastic cost-to-go function* $\mathcal {V}(z,\tau )$, that is the *minimum expected cost* that one needs to pay to go from $(z,\tau )$ to the end point subject to the constraints on the optimal control:

17 $$\begin{aligned} \mathcal {V}(z,\tau )= &  \mathbb {E} \left[ \min _{\begin{array}{c} |u(t)|\le u_{max} \\ \forall \, t \in [\tau ,T_{end}] \end{array}} J \right] \nonumber \\= &  \mathbb {E} \left[ \min _{\begin{array}{c} |u(t)|\le u_{max} \\ \forall \, t \in [\tau ,T_{end}] \end{array}} \left[ \int _{\tau }^{T_{end}} u^2 dt + \gamma q(z(T_{end})) \right] \right] .\nonumber \\ \end{aligned}$$

From Mitchell and Templeton (2005), the stochastic cost-to-go function satisfies the stochastic HJB equation (Fleming and Soner 2006; Yong and Zhou 1999):

18 $$\begin{aligned} \frac{\partial \mathcal {V}}{\partial t} + \min _{|u|\le u_{max}} \mathcal {H}(z,\nabla \mathcal {V},u) + \frac{D}{K^2} \frac{\partial ^2 \mathcal {V}}{\partial x^2}= &  0, \end{aligned}$$

where $\nabla \mathcal {V} \equiv (\frac{\partial \mathcal {V}}{\partial x},\frac{\partial \mathcal {V}}{\partial y})^T$ is the gradient of the value function with respect to *z*. The second-order term $\frac{D}{K^2} \frac{\partial ^2 \mathcal {V}}{\partial x^2}$ represents the stochastic effects in the system dynamics, where *D* is the noise intensity appearing in the stochastic HJB equation (18), and *K* is a scaling parameter introduced to address the different orders of magnitude between the voltage and gating variables, i.e., we take $x = V/K$. In our case, the Hamiltonian is Mitchell and Templeton (2005):

19 $$\begin{aligned} \begin{array}{l} \mathcal {H}(z,\nabla \mathcal {V},u) = u^2 + \nabla \mathcal {V}(z(t),t) (F(z(t)) + B u(t)). \end{array} \end{aligned}$$

Equation (18) is understood as backward in time, following the dynamic programming principle, where optimal controls are determined by reasoning from the desired final state (Bellman 1957; Bertsekas 1995; Pardoux and Peng 1990; El Karoui et al. 1997), and reduces to the deterministic HJB equation considered in Nabi et al. (2013) with solution $\mathcal {V} \equiv \mathcal {V}_d$ when $D=0$. The nonlinearity of the equation arises from the minimization term $\min _{|u|\le u_{max}} \mathcal {H}(z,\nabla \mathcal {V},u)$, which involves the Hamiltonian $\mathcal {H}(z,\nabla \mathcal {V},u)$ and the optimal control constraint $|u|\le u_{max}$.

As in Nabi et al. (2013), in order to find the optimal control we can set the derivative of the Hamiltonian (19) with respect to *u* equal to zero and solve for the extremal *u*, noting the possibility that it saturates at the bound $u_{max}$ in accordance with Pontryagin’s minimum principle (Pontryagin et al. 1962; Kirk 1970). This calculation results in:

20 $$\begin{aligned} \begin{array}{ll} u^*(t) = -\frac{1}{2K} \mathcal {V}_x & \;\; |\mathcal {V}_x| \le 2Ku_{max}, \\ u^*(t) = -\textrm{sign}(\mathcal {V}_x) u_{max} & \;\; |\mathcal {V}_x| > 2Ku_{max}, \end{array} \end{aligned}$$

where $\mathcal {V}_x = \frac{\partial \mathcal {V}}{\partial x}$. With this optimal control, the Hamiltonian can be written as

21 $$\begin{aligned} \begin{array}{ll} \mathcal {H} = \nabla \mathcal {V}^T F(z) -\frac{1}{4K^2} \mathcal {V}_x^2, & |\mathcal {V}_x| \le 2Ku_{max}, \\ \mathcal {H} = \nabla \mathcal {V}^T F(z) + u_{max}^2 - |\mathcal {V}_x| \frac{u_{max}}{K}, & |\mathcal {V}_x| > 2Ku_{max}. \end{array} \end{aligned}$$

We numerically solve the stochastic HJB equation (18) backward in time for $\mathcal {V}(z,t)$, using a Local Lax Friedrich WENO Hamilton–Jacobi solver for approximating the Hamiltonian and an implicit discretization of the stochastic term (Osher and Fedkiw 2003; Rajabi et al. 2024). We set $T_{end}=7$ ms and use a $320 \times 320$ uniform grid for the states. The control bound is set to be $u_{max}=10 \; \mathrm{\mu A/\mu F}$. We also set the end point cost to be:

22 $$\begin{aligned} \gamma q(z(T_{end})) = \gamma \left( 1-e^{- \left( \frac{(x-x_{pl})^2}{\sigma ^2_x} + \frac{(y-y_{pl})^2}{\sigma ^2_y} \right) }\right) , \end{aligned}$$

where $\gamma = 1000$, $\sigma ^2_x =\sigma ^2_y= 0.001$, and $(x_{pl},y_{pl})=(\frac{1}{K}V_{pl},n_{pl})$ where $K=100$ and $(V_{pl},n_{pl})=(-59.6,0.403)$ is the phaseless target point. This Gaussian end point cost function has a minimum of zero at the phaseless point that encourages the evolution of the controlled system toward this point. We solve the HJB equation backward in time and treat this end point cost as the initial condition for the equations, i.e.,

23 $$\begin{aligned} \mathcal {V}(z(T_{end}),T_{end}) = \gamma q(z(T_{end})). \end{aligned}$$

After computing the stochastic cost-to-go function $\mathcal {V}(z,t)$, we can find the optimal control using (20). This is done iteratively. Suppose we know that the trajectory is at a specific point in phase space at a specific time. From the value function $\mathcal {V}$, we use (20) to obtain the value of the control $u^*$ to be applied to (1) for one time step, also incorporating the effect of noise. This determines the new point for the trajectory in phase space. This process repeats to obtain the full trajectory and control input $u^*(t)$. Since the noise will affect the trajectory, we will obtain a different $u^*(t)$ for each realization of the stochastic trajectory. We call this approach to finding $u^*(t)$ evaluating the stochastic value function on the stochastic trajectory. It gives the energy-optimal control input for that particular stochastic trajectory. We also consider a variation where we compute the control input $\tilde{u}^*(t)$ by tracking the trajectory according to the solution to (1) with $D=0$, at each time step applying the appropriate $u^*(t)$ found from (20) but *without* incorporating the effect of noise on this trajectory. We do not expect $\tilde{u}^*(t)$ to be energy-optimal, but we can think of it as an approximation for the input that one would obtain when averaging over an ensemble of stochastic trajectories. We call this approach to finding $\tilde{u}^*(t)$ evaluating the stochastic value function on the deterministic trajectory. Since $\mathcal {V}(z,t)$ is only available on spatial grid points, in both of these approaches we use a simple bilinear interpolation scheme to obtain the input off grid points as needed (Press et al. 2007).

We solved the stochastic HJB equations numerically using a specialized computational framework that implements the numerical methods discussed above. Post-simulation analysis and visualization were conducted using MATLAB, which was used to generate the figures presented in this work.

**Fig. 3:**
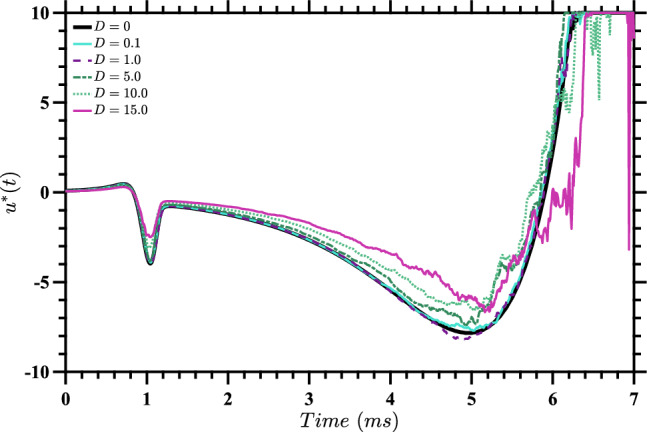
Time series of control inputs for stochastic trajectories. For each value of *D*, the optimal input $u^*(t)$ is calculated by evaluating the stochastic value function from (18) on stochastic trajectories for a single realization of noise. The input for $D=0$ is the same as the input which would be found using the approach in Nabi et al. (2013). The corresponding trajectories are shown in Fig. 4

**Fig. 4:**
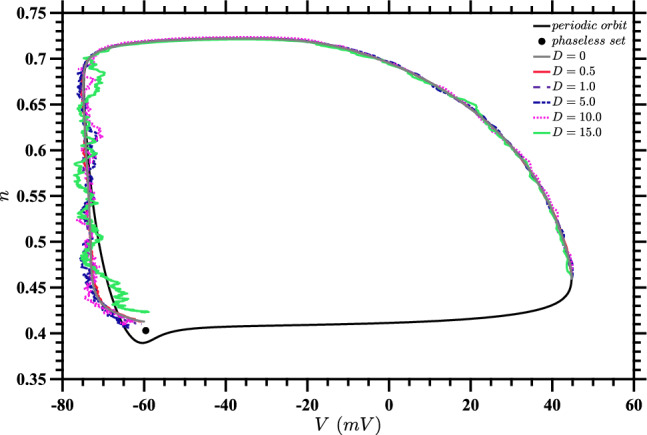
State-space diagram for the stochastic control of a single neuron oscillator under varying noise levels for a single noise realization. For each value of *D*, this shows the trajectory which arises from the inputs shown in Fig. 3. The plot also includes the periodic orbit and phaseless set

To investigate control of a single neuron, we consider (1). Figure 3 shows sample control inputs found by solving (18) for the stochastic value function for various values of *D*, and using (20) evaluated on the stochastic trajectories found from (1) to get the optimal $u^*(t)$; these trajectories are shown in Fig. 4. Despite rather large values of noise, the control is able to bring the trajectories close to the target point given by the phaseless set.

**Fig. 5:**
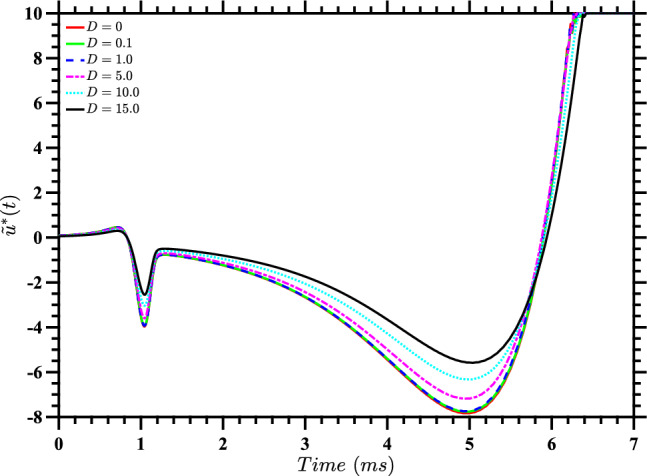
Control inputs $\tilde{u}^*(t)$ computed by evaluating the stochastic value function for each value of *D* along the corresponding deterministic trajectories, which are shown in Fig. 6. These nearly-optimal inputs are useful when only the value of *D* is known, and not the specific stochastic trajectory that is followed

**Fig. 6:**
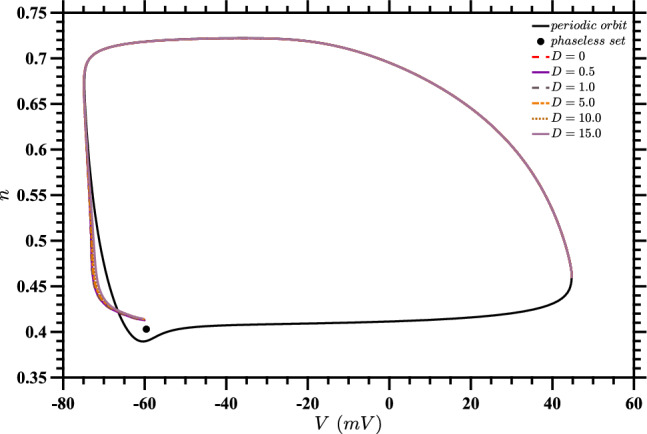
State-space diagram for stochastic value function evaluated on deterministic trajectories. This figure shows the trajectories obtained for a single deterministic neuron, using the inputs $u^*(t)$ from Fig. 5 for the corresponding values of *D*. The plot also shows the periodic orbit and phaseless set

Recognizing that it may not be possible to track the trajectory at all times in order to calculate the optimal $u^*(t)$ for a particular realization of noise, in Fig. 5 we show the control inputs $\tilde{u}^*(t)$ obtained by evaluating the stochastic value function from solving (18) at the deterministic trajectory obtained by solving (1) for a single neuron without noise (i.e., with $D=0$). The corresponding trajectories are shown in Fig. 6. We can think of these as inputs that one would use if only the value of *D* was known, and not the specific stochastic trajectory that was being followed. It is apparent from Fig. 5 that as *D* increases, the total input energy $\int [\tilde{u}^*(t)]^2 dt$ decreases.

We now compare the energy usage for three different control strategies: $u^*(t)$ which is obtained by evaluating the stochastic value function on the stochastic trajectory, $\tilde{u}^*(t)$ which is obtained by evaluating the stochastic value function on the deterministic trajectory, and $u_0^*(t)$ which is obtained by evaluating the deterministic value function (obtained from (18) with $D=0$) on the stochastic trajectory. Using $u_0^*(t)$ corresponds to using the value function obtained in the approach of Nabi et al. (2013), but evaluated on the specific stochastic trajectory of interest.

**Fig. 7:**
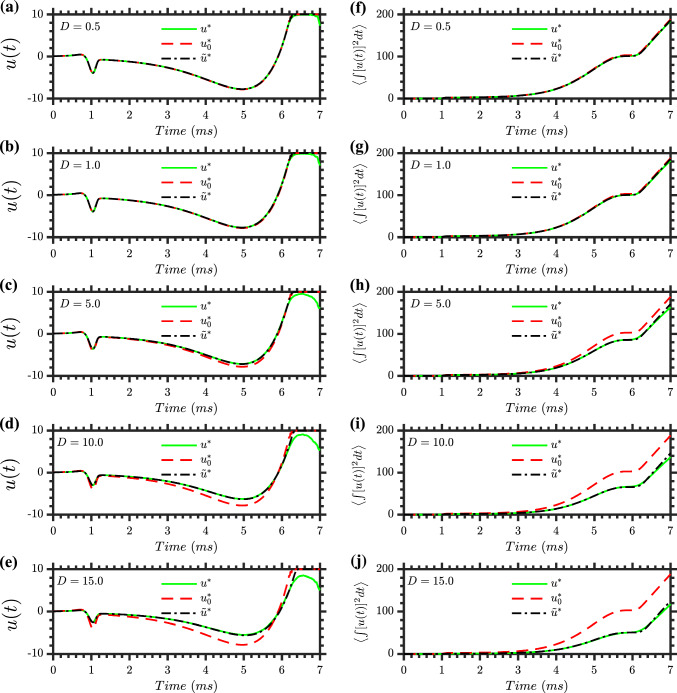
Control inputs and energy consumption across different noise levels. Results are based on 10,000 Monte Carlo simulations. Subplots **a**–**e** illustrate the control inputs over time for varying noise intensities *D*: the mean control input $\langle u^*(t) \rangle$ (stochastic optimal control applied to stochastic trajectories, green solid lines), the mean control input $\langle u_0^*(t) \rangle$ (deterministic optimal control applied to stochastic trajectories, red dashed lines), and $\tilde{u}^*$ (stochastic optimal control applied to deterministic trajectories, black dash-dotted lines). Subplots **f**–**j** display the corresponding mean energy consumption over time, represented by $\left\langle \int [u^*(t)]^2 dt \right\rangle$, $\left\langle \int u_0^*(t)]^2 dt \right\rangle$, and $\int [\tilde{u}^*(t)]^2 dt$. For the stochastic cases (green and red), the energy plots depict the mean value of the integral of squared control inputs, not the integral of the squared mean control inputs shown in **a**–**e**. This distinction is crucial, as $\left\langle \int [u^*(t)]^2 dt \right\rangle \ne \int [\left\langle u^*(t) \right\rangle ]^2 dt$ due to the nonlinearity of the squaring operation. These plots emphasize the comparative performance of different control strategies concerning control input behavior and energy efficiency across various stochastic realizations and noise levels

For $u^*(t)$ and $u_0^*(t)$, we considered 10,000 different realizations of the stochastic trajectories for each value of *D*, to better understand the mean energy usage for these strategies, and how this depends on noise level. Note that $\tilde{u}^*(t)$ does not depend on the specific stochastic trajectory. Figure 7 shows the results averaged over the 10,000 realizations: the left column shows the mean control input *u*(*t*), while the right column displays the mean cumulative energy consumption $\int u^2(t) dt$. These plots show that the inputs $u^*(t)$ and $\tilde{u}^*(t)$ use less total energy than the inputs $u_0^*(t)$. This is particularly evident for higher noise intensities. We can understand this phenomenon by noting that a positive *D* smooths out the value function via diffusion, so the derivative $\mathcal {V}_x$ will be smaller, and hence the magnitude of the control input found from (20) will typically be smaller. This illustrates that the input found by solving for the stochastic value function with the appropriate value for *D* has an energy advantage over using the deterministic value function, as was used in Nabi et al. (2013).

**Fig. 8:**
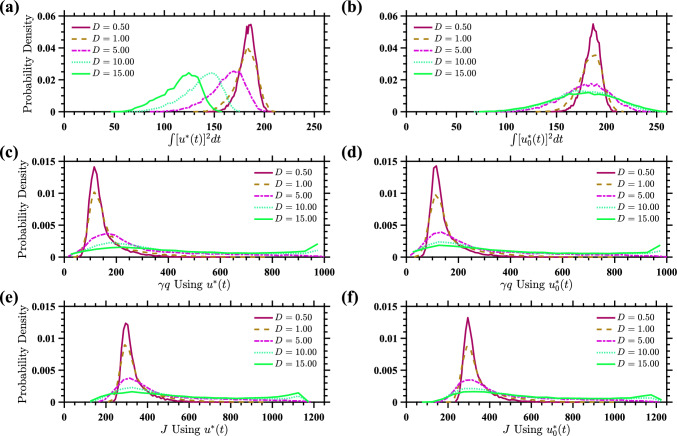
Probability distributions for control costs for stochastic ($u^*(t)$) and deterministic ($u_0^*(t)$) control strategies. Panels **a** and **b** illustrate the probability distributions of the total energy used, represented by the integral of the squared control input $\int [u^*(t)]^2 dt$, for stochastic and deterministic control strategies, respectively. Panels **c** and **d** depict the probability distributions of the final cost $\gamma q(T_{end})$ under both control strategies. Panels **e** and **f** present the distributions of the total cost *J* under both control strategies. The plots illustrate that stochastic and deterministic strategies have similar end-point and total cost distributions, but the stochastic strategy typically requires less energy and gives a total cost distribution with a slightly less extreme tail

**Fig. 9:**
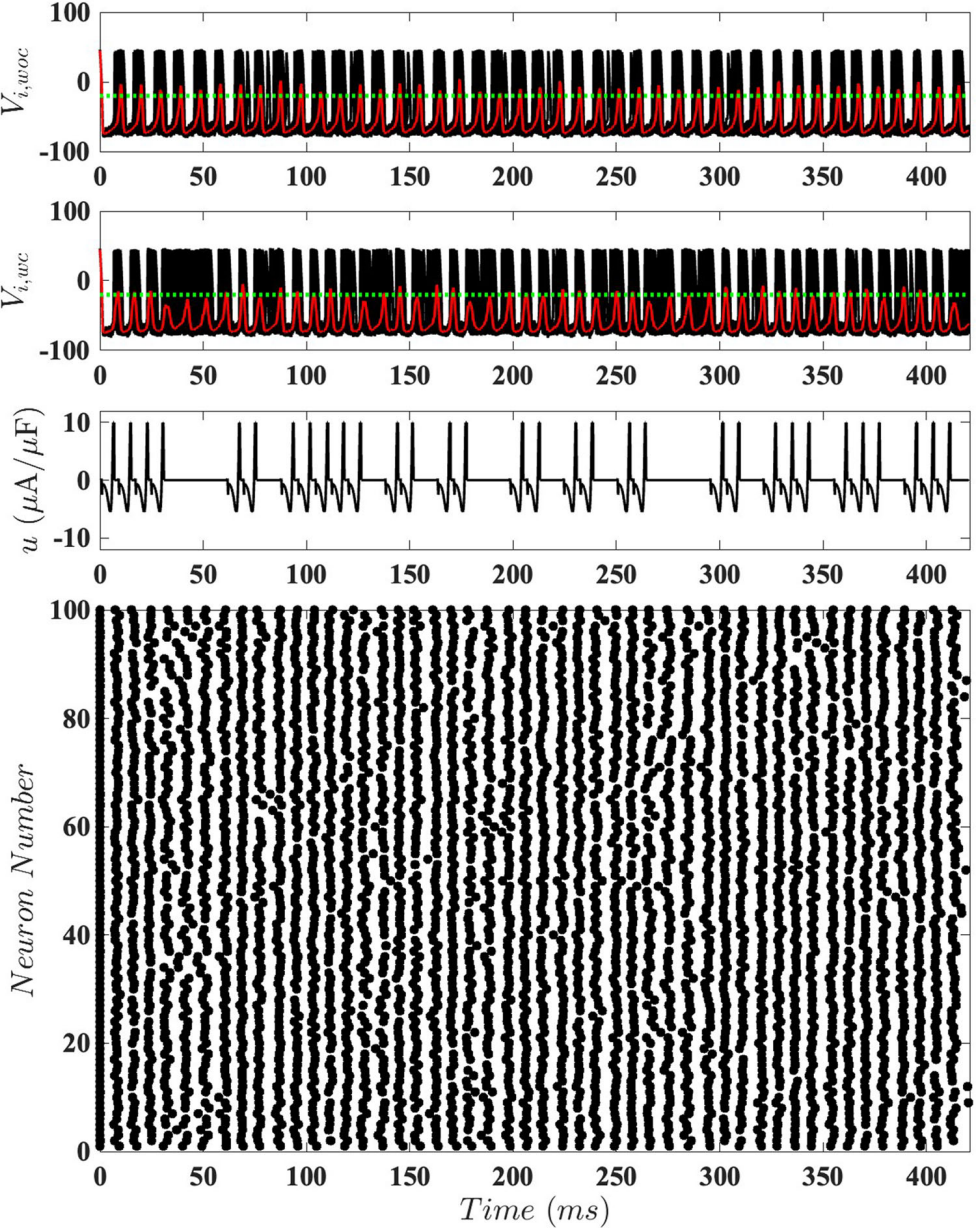
Results are shown for a population of $N = 100$ coupled neurons with $\eta _i = \sqrt{2D} N(0, 1)$, $D = 15$, and homogeneous coupling strength $\alpha _{ij} = 0.25$. The top panel displays the network’s behavior without control. The second panel illustrates the same network with active event-based control using $\tilde{u}^*(t)$, where the red traces represent the mean voltage for each case. The horizontal green dotted line indicates the control activation threshold ($\bar{V} = -20$ mV). The third panel presents the control input, and the bottom panel features a raster plot of spike times

**Fig. 10:**
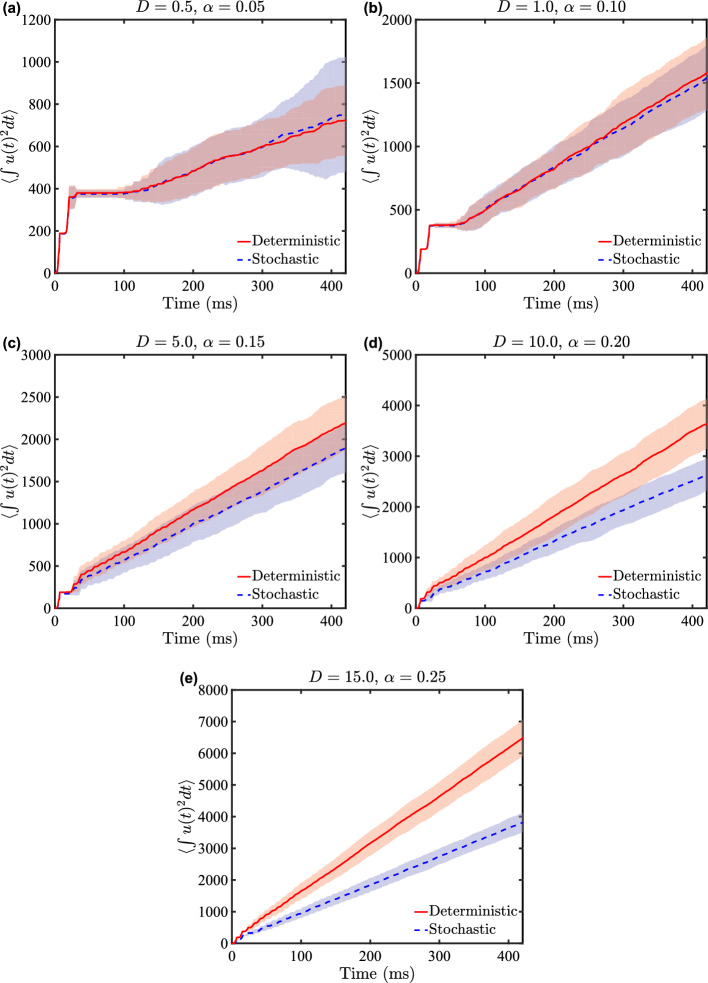
Comparison of mean cumulative energy expenditure for deterministic ($u_d^*(t)$) and stochastic ($\tilde{u}^*(t)$) optimal control strategies. Plots show $\int [u^*(t)]^2 dt$ averaged over 100 noise realizations, with variance indicated by the shaded regions, for different noise intensities *D* and corresponding coupling strengths $\alpha$. **a** $D = 0.5$, $\alpha = 0.05$; **b** $D = 1.0$, $\alpha = 0.1$; **c** $D = 5.0$, $\alpha = 0.15$; **d** $D = 10.0$, $\alpha = 0.2$; **e** $D = 15.0$, $\alpha = 0.25$. Red solid lines represent deterministic control (${u_d}^*(t)$), while blue dashed lines represent stochastic control ($\tilde{u}^*(t)$). These plots illustrate the comparative energy efficiency of deterministic and stochastic control strategies under varying conditions

**Fig. 11:**
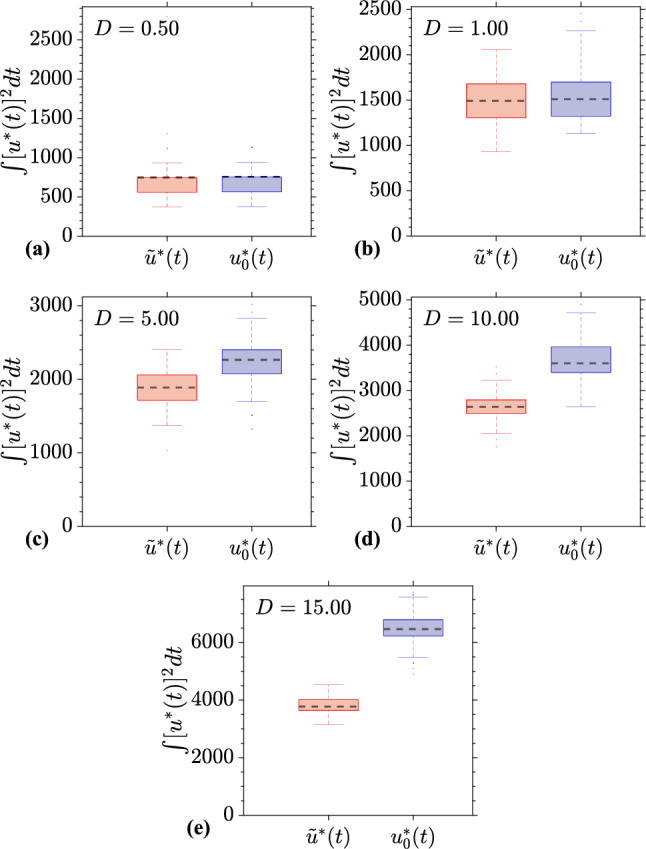
Distribution of cumulative energy expenditure for deterministic ($u_d^*(t)$) and stochastic ($\tilde{u}^*(t)$) optimal control strategies. Box plots show the distribution of $\int _0^{425} [u^*(t)]^2 dt$ for 100 different noise realizations, across different noise intensities *D* with corresponding coupling strengths $\alpha$ for each case. **a** $D = 0.5$, $\alpha = 0.05$; **b** $D = 1.0$, $\alpha = 0.1$; **c** $D = 5.0$, $\alpha = 0.15$; **d** $D = 10.0$, $\alpha = 0.2$; **e** $D = 15.0$, $\alpha = 0.25$. Red boxes represent the stochastic control strategy, while blue boxes represent the deterministic control strategy. For each box, the dashed line indicates the median, and the bottom and top edges of the box indicate the 25th and 75th percentiles, respectively. The whiskers extend to the most extreme data points not considered outliers, and the outliers are plotted individually using the ‘+’ symbol. These plots illustrate the variability and central tendencies of energy expenditure under different conditions for both control strategies

Finally, Fig. 8 compares the control performance between $u^*(t)$ and $u_0^*(t)$. In particular, it shows the probability distributions for the total input energy, the end-point cost $\gamma q$, and the total cost *J* given by (14), obtained from 10,000 Monte Carlo simulations over a 425 ms time interval for both control strategies. It is clear from panels (a) and (b) that the stochastic control $u^*(t)$ typically uses less energy than the deterministic control $u_0^*(t)$, particularly for larger values of *D*. This is consistent with the results shown in Fig. 7. The distributions for the end-point cost shown in panels (c) and (d) are quite similar, and exhibit long tails for larger values of *D*. The distributions for the total cost *J* are also quite similar, but we do notice that the distribution for the deterministic control $u_0^*$ has a longer tail than the distribution for the stochastic control $u^*(T)$. Overall, the stochastic and deterministic strategies have similar total cost distributions, but the stochastic strategy requires less energy and gives a total cost distribution with a slightly less extreme tail.

## Population-level control

We will now consider a population of coupled, noisy neurons described by the following set of equations:

24 $$\begin{aligned} \begin{aligned} \dot{V}_i&= f_V(V_i, n_i) + \frac{1}{N}\sum _{j=1}^{N} \alpha _{ij}(V_j - V_i) + u(t) + \eta _i(t), \\ \dot{n}_i&= f_n(V_i, n_i). \end{aligned} \end{aligned}$$

here $i = 1, \ldots , N$, where $N$ represents the total number of neurons in the network. The variables $V_i$ and $n_i$ denote the membrane voltage and gating variable for neuron $i$, respectively. As in Nabi et al. (2013), the coupling is assumed to be electrotonic, with strength between neurons $i$ and $j$ denoted by $\alpha _{ij}$. We assume that $\alpha _{ij} = \alpha _{ji}$ and $\alpha _{ii} = 0$ for all $i, j$. The intrinsic noise for each neuron, $\eta _i(t) = \sqrt{2D} \mathcal {N}(0,1)$, is modeled as zero-mean Gaussian white noise with variance $2D$. The common control input is $u(t) = I(t)/c$. The functions $f_V$ and $f_n$ and other functions and parameters are defined as above.

In this section, we will compare the new method from this paper (stochastic optimal control strategy) and the method used in Nabi et al. (2013) (deterministic optimal control strategy) for desynchronizing neural populations. Here our stochastic optimal control input will be $\tilde{u}^*(t)$, that is, the input obtained by evaluating the stochastic value function on the deterministic trajectory, and our deterministic optimal control input will be called $u_d^*(t)$, which is the input obtained by evaluating the deterministic value function on the deterministic trajectory, as was the approach used in Nabi et al. (2013). We emphasize that the control input is calculated for a single neuron, and is applied using an event-based strategy to the whole population. Although the coupling will provide a perturbation to each individual neuron’s trajectory, for the relatively weak coupling strengths considered in this paper we expect that to leading order the effect of the control input on a coupled neuron will be very similar to the effect of the control input on a single, uncoupled neuron.

### Network with homogeneous coupling

We consider a network of $N = 100$ coupled initially-synchronized noisy neurons. The network is characterized by a common coupling strenth $\alpha _{ij} = \alpha$ and independent and identically distributed (i.i.d.) noise with intensity *D*. It is useful to think of noise as a desynchronizing influence, and coupling as a synchronizing influence. If $\alpha$ is fixed, increasing *D* will make it easier to desynchronize the population; on the other hand, if *D* is fixed, increasing $\alpha$ will make it more difficult to desynchronize the population. In the following, for each noise level $D \in \{0.5, 1.0, 5.0, 10.0, 15.0\}$ used in the HJB equation, we use a corresponding coupling strength $\alpha \in \{0.05, 0.1, 0.15, 0.2, 0.25\}$ for the neuron population, respectively. These values are chosen as a sampling of different points in parameter space.

We implement an event-based control strategy as follows. Define the mean voltage as the observable for the network:

25 $$\begin{aligned} \bar{V}(t) = \frac{1}{N} \sum _{i=1}^N V_i(t), \end{aligned}$$

where $V_i(t)$ is the voltage of the *i*-th neuron at time *t*. We set $\bar{V} = -20$ mV as the event threshold that triggers one 7 ms cycle of control $\tilde{u}^*(t)$ (which was found by evaluating the stochastic value function on the deterministic trajectory, calculated for the single neuron model). After one cycle, the control is deactivated until the next threshold crossing event occurs. The same control input $\tilde{u}^*(t)$ is used for each threshold crossing.

Figure 9 illustrates this event-based control strategy using stochastic optimal control for $D=15$ and $\alpha =0.25$. For our chosen $(D,\alpha )$ pairs, as *D* increases the tendency for the coupling to synchronize the neurons becomes relatively stronger than the tendency for noise to desynchronize them; thus, more control inputs, and hence more energy, will be needed to keep the neurons desynchronized.

A comparative analysis of deterministic and stochastic optimal control strategies reveals intriguing dynamics in neural population management for varying noise intensities. Figure 10 illustrates the cumulative energy expenditure, quantified by $\int [u^*(t)]^2 dt$, for both control strategies under different noise conditions (*D*) and coupling strengths ($\alpha$), averaged over 100 independent realizations. We see that the stochastic optimal control strategy demonstrates superior energy efficiency compared to its deterministic counterpart, in particular for $(D,\alpha ) = (10,0.2)$ and $(D,\alpha ) = (15,0.25)$. This efficiency differential manifests as a progressive divergence between the energy expenditure curves of the two strategies. In particular, the solid lines representing deterministic control consistently lie above the dashed lines of stochastic control, with this separation becoming more pronounced over time. The temporal onset of this divergence occurs earlier, and its magnitude grows more rapidly, as we progress through subplots (a) to (e). As an example quantifying the energy savings, when $(D,\alpha ) = (15,0.25)$, at $T=425$ the average cumulative energy for stochastic control is 3800 for stochastic control, whereas for deterministic control it is 6500. This represents $32\%$ average reduction in the energy needed to desynchronize the population.

Figure 10 also offers additional insights. For $(D,\alpha ) = (0.5, 0.05)$ and $(D,\alpha ) = (1.0,0.1)$, we observe a nuanced three-phase pattern in the energy expenditure curves. Initially, there’s a steep climb, attributable to the energy required to break the strong initial synchronization of neurons. This is followed by a brief plateau, suggesting a period of minimal necessary control, before transitioning to a phase characterized by a roughly constant positive slope. This final phase likely represents the averaged effect of varied noise-induced trajectories. Interestingly, this three-phase pattern becomes less distinct for $(D,\alpha ) = (10,0.2)$ and $(D,\alpha ) = (15, 0.25)$, where the system’s behavior is smoothed out more rapidly.

The better performance of the stochastic control strategy, especially for $(D,\alpha ) = (10,0.2)$ and $(D,\alpha ) = (15,0.25)$ and over extended time periods, suggests that it possesses a powerful capability to leverage the system’s intrinsic noise characteristics. This advantage likely stems from the strategy’s foundation in the stochastic HJB equation, which allows for a more energy-efficient approach to neural population control.

**Fig. 12:**
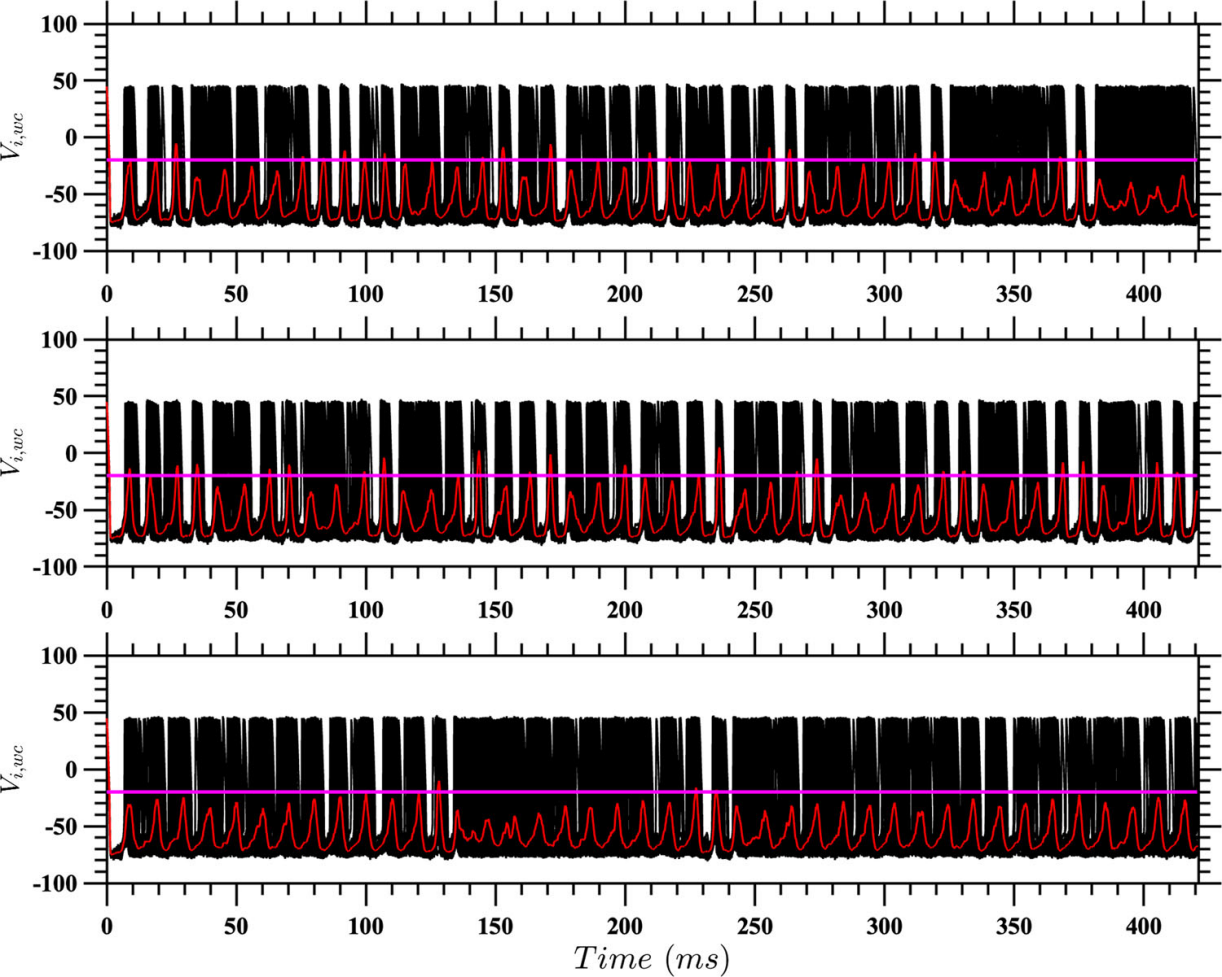
Neural network behavior under varying coupling strength conditions with active event-based control for $D = 10$. Results are presented for a population of $N = 100$ coupled neurons with active event-based control using $\tilde{u}^*(t)$ and noise characterized by $\eta _i = \sqrt{2D}\mathcal {N}(0,1)$, where $D = 10$. The panels show different coupling strength scenarios. Top panel: The network has a uniform coupling strength $\bar{\alpha } = 0.20$; Middle pane: The coupling strengths are drawn from the distribution described in the text, with a standard deviation of 20% of the mean coupling strength; Bottom panel: The coupling strengths are the same as in the middle panel, with the additional modification that $20\%$ of the coupling strengths are randomly set to zero. To facilitate comparison, the random number generators were seeded to produce the same values for the random vectors across all three experiments. The red traces represent the mean voltage for each case, and the horizontal pink lines indicate the control activation threshold. The control is activated only when the mean voltage exceeds this threshold

**Fig. 13:**
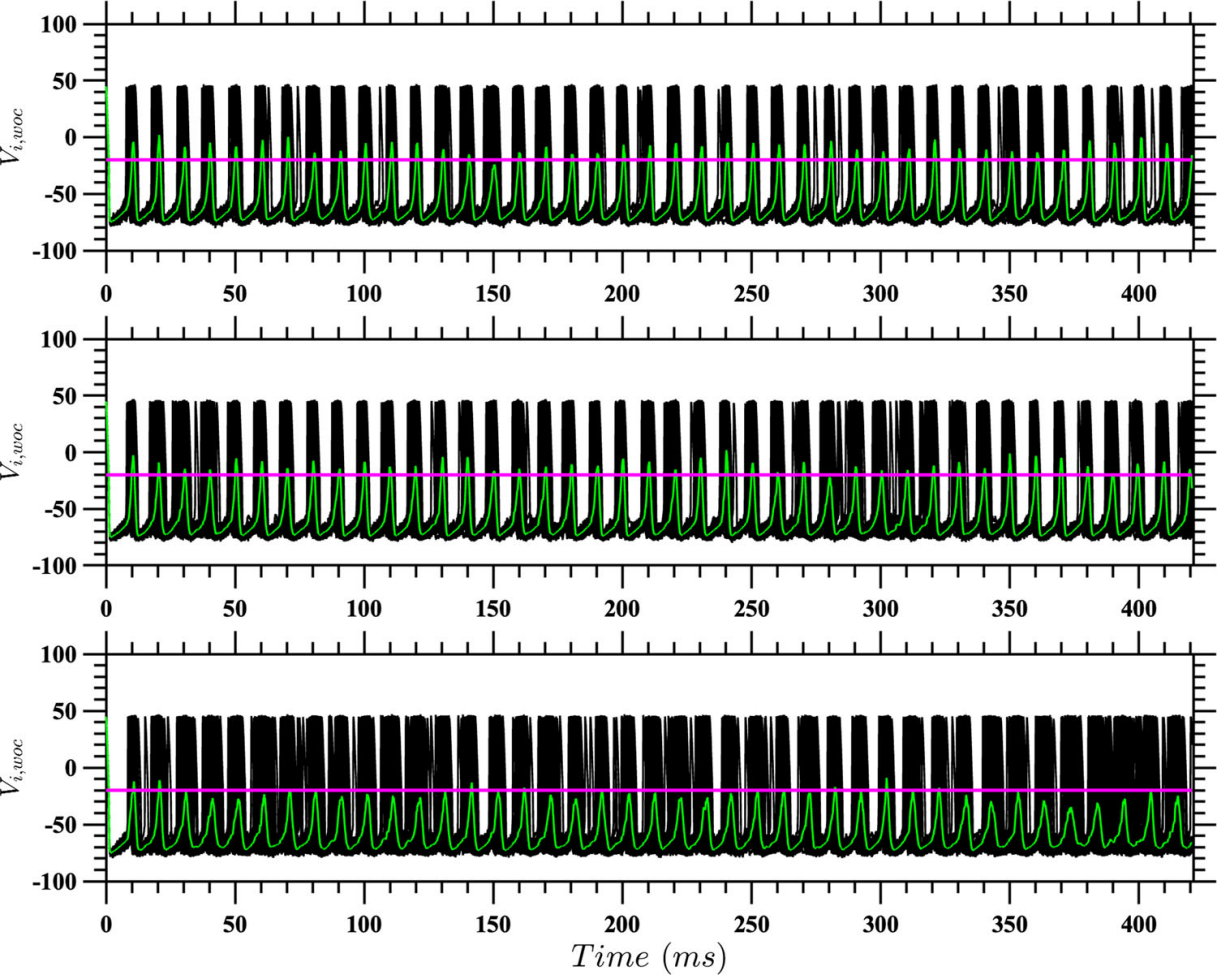
Neural network behavior under varying coupling strength conditions without active event-based control for $D = 10$. Results are presented for a population of $N = 100$ coupled neurons with noise characterized by $\eta _i = \sqrt{2D}\mathcal {N}(0,1)$, where $D = 10$. The panels show different coupling strength scenarios. Top panel: The network has a uniform coupling strength $\bar{\alpha } = 0.20$; Middle panel: The coupling strengths are drawn from the distribution described in the text, with a standard deviation of 20% of the mean coupling strength; Bottom panel: The coupling strengths are the same as in the middle panel, with the additional modification that $20\%$ of the coupling strengths are randomly set to zero. To facilitate comparison, the random number generators were seeded to produce the same values for the random vectors across all three experiments. The green traces represent the mean voltage for each case, and the horizontal pink lines indicate the control activation threshold. The control is absent, allowing natural network dynamics to unfold

Finally, we consider statistics of the cumulative energy used, defined as $\int _0^T [u^*(t)]^2 dt$ for $T = 425$ ms, for both the stochastic and deterministic cases. This quantifies the total control effort required to achieve the desired state transition in the neuron population. Figure 11 gives statistics for this for different $(D,\alpha )$ pairs. This illustrates the variability in energy expenditure across different trials, and further demonstrates that the stochastic optimal control strategy desynchronizes neural populations using less energy than the deterministic optimal control strategy, particularly for $(D,\alpha ) = (10,0.2)$ and $(D,\alpha ) = (15,0.25)$.

### Robustness to network properties

To assess the robustness of our control strategy to different networks, we performed an analysis across various coupling strength scenarios and noise levels. This investigation introduces heterogeneities into the network, providing key insights into the applicability and efficiency of our approach under diverse conditions. We explored five distinct noise levels, $D \in \{0.5, 1, 5, 10, 15\}$, each paired with a corresponding mean coupling strength $\bar{\alpha } \in \{0.05, 0.10, 0.15, 0.20, 0.25\}$, respectively. For each noise level, we examined three network configurations: (1) a homogeneous network with uniform coupling strength $\alpha _{ij} = \bar{\alpha }$, which is equivalent to the analysis above in Sect. 4.1, (2) a heterogeneous network with coupling strengths drawn from a normal distribution $\alpha _{ij} = \alpha _{ji} \sim \mathcal {N}(\bar{\alpha }, 0.2\bar{\alpha })$, and (3) a sparse heterogeneous network where $20\%$ of randomly chosen connections were set to zero. This design allows us to systematically study the effects of coupling strength variability and network sparsity on control performance.

For each configuration and noise level, we simulated a population of 100 coupled neurons under active event-based control, where we again use $\tilde{u}^*$ as the control input when the average voltage crosses the threshold. The intrinsic noise for each neuron was modeled as $\eta _i = \sqrt{2D}\mathcal {N}(0,1)$, representing neural stochasticity. To ensure statistical robustness, 100 independent realizations were performed for each scenario. Results for one such realization with $D=10$ and $\bar{\alpha } = 0.20$ are shown in Fig. 12. Here the three panels represent the three network configurations: homogeneous, heterogeneous, and sparse heterogeneous networks. In each panel, the red dotted traces represent the mean voltage, while the horizontal dotted lines indicate the control activation threshold. Control is applied only when the mean voltage exceeds this threshold. For comparison, corresponding results without control are shown in Fig. 13.

**Fig. 14:**
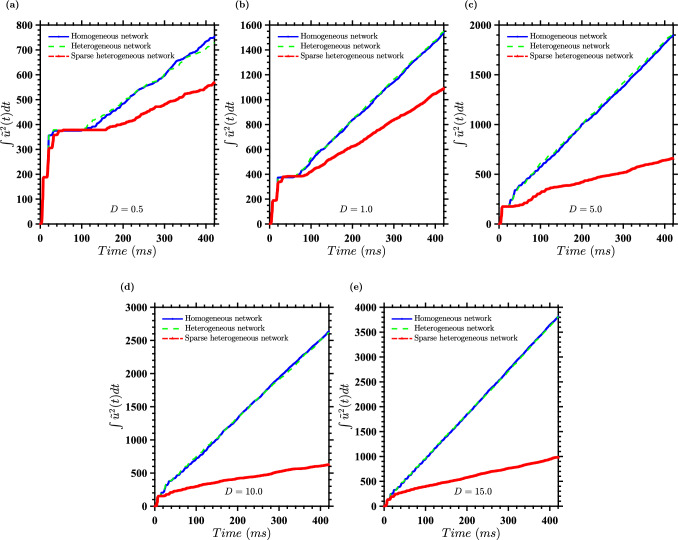
Robustness analysis of the mean cumulative energy expenditure for different coupling scenarios. Plots show $\int [u^*(t)]^2 dt$, averaged over 100 realizations, across different noise intensities *D* and coupling scenarios with corresponding coupling strengths $\alpha _{ij}$ for each case. **a** $D = 0.5$, $\bar{\alpha } = 0.05$; **b** $D = 1.0$, $\bar{\alpha } = 0.1$; **c** $D = 5.0$, $\bar{\alpha } = 0.15$; **d** $D = 10.0$, $\bar{\alpha } = 0.2$; **e** $D = 15.0$, $\bar{\alpha } = 0.25$. Blue lines represent uniform coupling ($\alpha _{ij} = \bar{\alpha }$), green lines represent normally distributed coupling ($\alpha _{ij} \sim \mathcal {N}(\bar{\alpha }, 0.2 \bar{\alpha })$), and red lines represent partially decoupled scenarios (80% of population with $\alpha _{ij} \sim \mathcal {N}(\bar{\alpha }, 0.2 \bar{\alpha })$, 20% with $\alpha _{ij} = 0$). These plots illustrate the robustness of the control strategy under different coupling conditions and noise intensities

As in Sect. 4.1, for homogeneous coupling and our chosen $(D,\bar{\alpha })$ pairs, as *D* increases the tendency for the coupling to synchronize the neurons becomes relatively stronger than the tendency for noise to desynchronize them. Therefore, more energy is needed to keep the neurons desynchronized. From Fig. 14 for energy expenditure (also see Table 1) over 425 ms intervals, we see that this is also the case for heterogeneous networks. In fact, the energy statistics for the homogeneous and heterogeneous networks are quite similar. In contrast, the energy and duration statistics for the sparse heterogeneous networks do not follow the same pattern, with the mean energy being lower for $(D,\bar{\alpha }) = (5,0.15)$ and $(D,\bar{\alpha }) = (10.0,0.20)$ than for $(D,\alpha ) = (1,0.10)$.

Interestingly, for $(D,\bar{\alpha }) = (0.5, 0.05)$ and $(D,\bar{\alpha }) = (1.0,0.1)$, differences in energy expenditure between the network types are relatively small. However, significant divergence occurs for $(D,\bar{\alpha }) = (5.0,0.15)$, where sparse heterogeneous networks require considerably lower energy expenditure (754.88 vs. 1918.11 in homogeneous and heterogeneous cases). For $(D,\bar{\alpha }) = (15.0,0.25)$, sparse heterogeneous networks require only about $26.5\%$ of the energy (1012.22 vs. approximately 3815.53) compared to homogeneous and heterogeneous networks. This increasing efficiency for control of sparse heterogeneous networks appears to be due to a delay in resynchronization for the sparse heterogeneous networks, so fewer control inputs are necessary. This delay is apparent in Fig. 12, and we hypothesize that it is due to the weakened tendency toward synchronization caused by broken connections.

**Table 1:** Comparison of energy consumption for different noise intensities and network types

Homogeneous		Heterogeneous	Sparse heterogeneous
(*D*, $\bar{\alpha }$)	$\left\langle \int \tilde{u}^2(t) dt \right\rangle \pm \sigma$	$\left\langle \int \tilde{u}^2(t) dt \right\rangle \pm \sigma$	$\left\langle \int \tilde{u}^2(t) dt \right\rangle \pm \sigma$
(0.5, 0.05)	752.97 ± 271.03	729.17 ± 171.87	546.85 ± 103.62
(1.0, 0.10)	1540.44 ± 260.15	1548.44 ± 244.14	1048.92 ± 222.71
(5.0, 0.15)	1895.18 ± 290.00	1918.11 ± 348.47	754.88 ± 201.24
(10.0, 0.20)	2636.17 ± 323.91	2619.58 ± 284.80	646.08 ± 247.77
(15.0, 0.25)	3815.53 ± 304.46	3813.70 ± 286.43	1012.22 ± 289.43

Results show $\left\langle \int \tilde{u}^2(t) dt \right\rangle \pm \sigma$ (mean ± standard deviation of energy consumption, $(\mu A/\mu F)^2 \cdot ms$) for $N = 100$ coupled neurons with event-based control, for the total time interval of 425 ms. Noise is $\eta _i = \sqrt{2D}\mathcal {N}(0,1)$, where *D* is noise intensity. $\bar{\alpha }$ is mean coupling strength. Network types: Homogeneous ($\alpha _{ij} = \bar{\alpha }$), Heterogeneous ($\alpha _{ij} \sim \mathcal {N}(\bar{\alpha }, 0.2\bar{\alpha })$), Sparse Heterogeneous (80% $\alpha _{ij} \sim \mathcal {N}(\bar{\alpha }, 0.2\bar{\alpha })$, 20% $\alpha _{ij} = 0$). $\tilde{u}$ represents stochastic HJB solution on deterministic trajectories. The data is averaged over 100 noise realizations

## Conclusion

In this study, we found energy-optimal control inputs for phase resetting for stochastic neural oscillators, which generalizes our earlier work on optimal phase resetting for deterministic neural oscillators (Nabi et al. 2013). This was accomplished by designing and implementing a nonlinear second-order monotone scheme solver for Backward Stochastic Partial Differential Equations (BSPDEs), which was applied to solving the stochastic Hamilton–Jacobi–Bellman (HJB) equation using level set methods. The optimal inputs were used to desynchronize populations of neural oscillators through an event-based feedback control approach, where control is activated whenever the average voltage of the neuronal population exceeds a given threshold.

Our results demonstrate that the stochastic optimal control strategy can outperfor its deterministic counterpart in terms of energy efficiency, especially in high-noise environments. While we have only demonstrated this for a particular neuron model, a particular type of coupling, and specific values of the noise and coupling strengths, we postulate that using the stochastic optimal control strategy will typically outperform the deterministic strategy for other models and types of coupling as well. This improvement could help to extend the battery life of implanted stimulus generators used in Deep Brain Stimulation (DBS) for Parkinson’s disease. For the coupling and noise strengths considered, it was found that much less energy is needed to desynchronize heterogenous neural networks, because control is less frequently active.

These findings could carry significant implications for neurostimulation and the treatment of neurological disorders characterized by pathological synchronization, such as Parkinson’s disease. By incorporating noise into control strategies, we have developed a more energy-efficient and potentially more effective method for neural modulation. Our work underscores the importance of considering noise in optimal control problems for physiological systems. Revisiting previous studies on seizure-like bursting (Wilson and Moehlis 2014a) and cardiac arrhythmias (Wilson and Moehlis 2014b) with noise taken into account could yield valuable new insights. Moreover, this computational framework could be applied to other domains where stochastic optimal control problems are prevalent, including robotics (Blackmore et al. 2011; Lioutikov et al. 2014), aerospace (Yao 2020; Bansal et al. 2017), and finance (Mnif 2007; Bielecki et al. 2005; Nagai 2021; Bo and Wang 2017). Recent work has also demonstrated the power of HJB methods in quantum control applications (Fromonteil et al. 2024).

A promising future research direction would be to focus on refining the BSPDE solver and applying it to more complex, higher-dimensional neural models. Translating these theoretical findings into practical applications, such as adaptive DBS protocols that account for the inherent stochasticity of neural systems, is also of great interest. Furthermore, investigating the long-term effects of these control strategies on neural plasticity and network reorganization, as well as conducting comparative clinical studies, will be crucial for optimizing therapeutic interventions and validating the real-world benefits of our stochastic control approach.

## Data Availability

The MATLAB post-processing code and simulation data used in this study are available at https://github.com/UCSB-CASL/HH-Stochastic-Control (DOI: 10.5281/zenodo.14015566). The repository includes processing and visualization scripts, HJB solution data for various noise levels, and documentation. The code requires MATLAB R2020a or newer with the Statistics and Signal Processing Toolbox and Optimization Toolbox. The provided code and data allow for the reproduction of all figures in the paper.
